# Predictors of change in sleep disturbance in Canadian long-term care facilities: a longitudinal analysis based on interRAI assessments

**DOI:** 10.1007/s41999-025-01302-z

**Published:** 2025-09-10

**Authors:** Nasir Wabe, Lisa Geyskens, Jae Yoon Yi, Edoardo Varratta, Alcina Matos Queirós, Luke Andrew Turcotte, Andrew Costa, John P. Hirdes

**Affiliations:** 1 Australian Institute of Health Innovation, Macquarie University, Sydney, Australia; 2 Department of Public Health and Primary Care, Gerontology and Geriatrics, KU Leuven, Louvain, Belgium; 3 Research Foundation–Flanders (FWO), Brussels, Belgium; 4 Department of Public Health Sciences, Graduate School of Public Health, Seoul National University, Seoul, Korea; 5 Università Cattolica del Sacro Cuore, Rome, Italy; 6 Department of Health and Social Welfare, Vaud, 1018 Lausanne, Switzerland; 7 Department of Health Sciences, Brock University, St. Catharines, ON Canada; 8 Faculty of Health Sciences, McMaster University, Hamilton, Canada; 9 School of Public Health Sciences, University of Waterloo, Waterloo, Canada

**Keywords:** Assessment, Quality of care, Care planning, Outcomes, Performance measurement

## Abstract

**Purpose:**

Sleep disturbance is prevalent in long-term care facilities (LTCFs), yet there is limited understanding of individual factors predicting changes in sleep within these populations. Our objective was to determine predictors of sleep disturbance in LTCFs and investigate variation in prevalence across facilities in two Canadian provinces—New Brunswick and Saskatchewan.

**Method:**

This retrospective longitudinal cohort study used interRAI comprehensive health assessment data from 2016 to 2021, encompassing 21,394 older adults aged ≥ 65 years across 228 LTCFs. Generalised estimating equations were used to determine predictors of sleep disturbance, with separate models for new and resolved sleep disturbance. Funnel plots were employed to assess facility-level variation, with facilities exceeding the 99.8th percentile control limit identified as outliers.

**Results:**

The overall prevalence of sleep disturbance was 21.7%, with rates ranging from 3 to 56% across facilities, and 8% of facilities showing outlying rates. Nine predictors were significantly associated with the onset of new sleep disturbance, including being a male, being newly admitted, cognitive impairment, pain, daytime sleeping, chronic obstructive pulmonary disease, coronary heart disease, antipsychotics use, and sedative-hypnotics use. Significant predictors of resolved sleep disturbance were stroke, polypharmacy, and being newly admitted. Conversely, lower odds of resolved sleep disturbance were observed among daytime sleepers and residents taking sedative-hypnotics.

**Conclusion:**

This study underscores the high prevalence and variation of sleep disturbance in LTCFs, highlighting potential modifiable risk factors for improvement. Further research is needed to explore the interplay of institutional, environmental, and individual factors to develop targeted interventions that enhance the quality of care in LTCFs.

**Supplementary Information:**

The online version contains supplementary material available at 10.1007/s41999-025-01302-z.

## Introduction

Sleep is a fundamental biological process essential for maintaining physical and mental health. It facilitates vital functions such as memory consolidation, emotional regulation, and immune system maintenance, contributing to overall well-being [1]. Despite its importance, the quality of sleep tends to decline with age due to physiological changes in sleep patterns, including reduced time in deep sleep and more frequent awakenings [2]. These age-related changes are often exacerbated by chronic illnesses, polypharmacy, and environmental factors, making older adults particularly vulnerable to sleep disturbance [3].

Sleep disturbance, characterised by difficulty in initiating or maintaining sleep, is highly prevalent among older adults, especially those living in long-term care facilities (LTCFs). A meta-analysis showed that 20% of older adults in LTCFs with dementia experience clinically significant sleep problems [4]. This prevalence is likely influenced by individual-level characteristics such as multimorbidity, cognitive impairment and chronic pain, as well as facility-specific environmental factors, including noise, lighting, and staff routines.

The consequences of sleep disturbance in the ageing population extend far beyond disrupted rest. At the individual level, poor sleep is strongly associated with adverse health outcomes, including an increased risk of cardiovascular diseases, cognitive decline, and depression [5]. Sleep disturbance is also linked to diminished quality of life, as they impair daily functioning, exacerbate existing health conditions, and reduce social engagement [6]. Moreover, sleep problems often trigger the use of psychotropic medications, which can increase the risk of falls, delirium, and other adverse events in older adults [4].

These issues are not confined to the affected individuals; they also have important implications for caregivers and healthcare systems. For instance, sleep disturbance in LTCF residents contributes to heightened stress and burnout among staff, affecting the quality of care provided [4]. Addressing these issues is therefore critical not only for improving individual health outcomes but also for reducing challenges for caregivers and optimising resource use in LTCFs.

Despite the clear need, there is a limited understanding of the individual factors that predict changes in sleep disturbance in LTCF populations. Identifying these predictors is essential for designing effective, evidence-based interventions to manage and improve sleep quality in this vulnerable group. Moreover, the ability to manage sleep disturbances effectively may be informative as an indicator of quality of care in LTCFs. Research that sheds light on these dynamics can support targeted strategies to enhance residents’ quality of life and reduce the reliance on pharmacological treatments. This study aims to fill this critical knowledge gap by leveraging interRAI comprehensive health assessment data to: (1) estimate the prevalence of sleep disturbance and identify predictors of both new and resolved in sleep disturbance over time in LTCFs and (2) examine variation in the prevalence of sleep disturbance across different LTCFs.

## Methodology

### Study setting and design

We conducted a retrospective longitudinal cohort study using comprehensive clinical assessment records extracted from 228 LTCFs across two Canadian Provinces: New Brunswick and Saskatchewan. The study data covered the period from July 13, 2016, to December 30, 2021, for New Brunswick, while data from Saskatchewan were available from July 1, 2019, to December 29, 2021. Research ethics board approval to perform secondary analysis was provided by the University of Waterloo (ORE #: 30173). In compliance with the Tri-Council Policy Statement: Ethical Conduct for Research Involving Humans—TCPS 2 (2022), Article 5.5B, informed consent was not required, as the study involved only the secondary analysis of fully anonymised and non-identifiable routinely collected electronic health data [7]. This paper was structured following the REporting of studies Conducted using Observational Routinely-collected health Data (RECORD) statement [8].

### Data sources

We used interRAI Long-term Care Facility (interRAI LTCF) records from the Canadian Institute for Health Information’s (CIHI) Integrated interRAI Reporting System (IRRS). The interRAI LTCF is a comprehensive health assessment that is used to collect standardised data across a numerous domains including demographics (e.g. age, gender), disease diagnoses (e.g. dementia, Parkinson’s disease, depression), symptoms and functional status (e.g. incontinence, daytime sleeping, sleep disturbance, fatigue, vision and hearing impairments, cognition, activities of daily living, social engagement), and treatments (e.g. number of medications/polypharmacy, use of antipsychotics). Additionally, information from these assessments is used to score validated outcome scales (e.g. the Changes in Health, End-Stage Disease, and Signs and Symptoms [CHESS] Scale, Pain Scale) and care planning algorithms (e.g. Communication Clinical Assessment Protocol).

### Study cohort

Supplementary Figure S1 illustrates the participant selection flowchart. The study cohort consisted of older adults aged 65 years and above who had at least two data points: the initial assessment at Time 1 (T1) and a follow-up assessment at Time 2 (T2), conducted three months later. The T1 assessment was the first assessment conducted as part of admission into the LTCF, while the T2 assessment was a routine reassessment. Individuals with only one assessment were excluded, as the study focused on examining longitudinal changes in outcomes over time, specifically between T1 and T2.

### Outcome measure

The outcome measure was a change in sleep disturbance. A sleep disturbance was measured using the following item in the interRAI LTCF: “Difficulty falling or staying asleep, waking up too early, restlessness and/or non-restful sleep”, with the response options: “Not present” (score of 0); Present but not exhibited in last 3 days (score of 1); Exhibited on 1 of last 3 days (score of 2); Exhibited on 2 of last 3 days (score of 3); and Exhibited daily in last 3 days (score of 4). Residents with a score of 1 or more were deemed as having sleep disturbance. In this study, change in sleep disturbance was evaluated based on two patterns: new sleep disturbance, defined as the absence of sleep disturbance at T1 followed by its presence at T2, and resolved sleep disturbance, defined as the presence of sleep disturbance at T1 followed by its absence at T2.

### Independent variables

We considered a comprehensive range of potential independent variables available in the interRAI LTCF assessment. The selection process was guided by experienced researchers familiar with similar datasets and informed by a literature review identifying common risk factors associated with sleep disturbance in LTCFs. The independent variables included socio-demographics (age categorised as 65–74, 75–84, and ≥ 85 years; gender; and length of stay in the facility, classified as < 1 year or ≥ 1 year), disease diagnoses (dementia, Parkinson’s disease, depression, chronic obstructive pulmonary disease, stroke, coronary heart disease, congestive heart failure), symptoms and functional status (blader and bowel incontinence, pain, daytime sleeping, fatigue, vision and hearing impairments), treatments (e.g. polypharmacy, use of antipsychotics, sedative-hypnotics), and several validated scales such as the Activities of Daily Living Hierarchy Scale (ADL-H) [9], Cognitive Performance Scale (CPS) [10], and the Revised Index of Social Engagement (RISE) [11].

### Statistical analysis

We reported descriptive statistics, including medians with interquartile ranges (IQR), as appropriate. The Chi-square test was used to compare the distribution of baseline characteristics between individuals with and without sleep disturbance at baseline. To identify longitudinal predictors of changes in sleep disturbance, we employed generalised estimating equations (GEE) with a binomial distribution and a logit link function, accounting for clustering effects at the facility level. Two separate GEE models were fitted by dividing the dataset into sub-samples based on sleep disturbance status at T1: model 1 examined new sleep disturbance (no sleep disturbance at T1 → sleep disturbance at T2), and model 2 focused on resolved sleep disturbance (sleep disturbance at T1 → no sleep disturbance at T2). The strength of associations was expressed as odds ratios (OR) with 95% confidence intervals (CIs).

We employed funnel plots to visualise the variation in the distribution of sleep disturbance across facilities. Funnel plots, a statistical process control tool, are widely used to compare institutional performance on an indicator of interest against a benchmark, to identify outlying practices [12]. In this study, the funnel plots were generated by plotting the prevalence of sleep disturbance for each facility (as a scatterplot) against the facility size (number of residents) on the x-axis. To facilitate interpretation, 95% and 99.8% control limits were superimposed around the benchmark, which was the overall prevalence of sleep disturbance, to identify facilities with potential outlying practices. All statistical tests were two-tailed, with significance set at P < 0.05. Statistical analyses were performed using SAS software (version 9.4, SAS Institute Inc., Cary, NC, USA).

## Results

### Participants

The study sample included 21,394 residents, of whom 78.3% (*n* = 16,749) did not have sleep disturbance at T1 and were included in model 1, while 22.7% (*n* = 4645) had sleep disturbance and were included in model 2 (Figure S1). interRAI assessments are completed on all persons admitted to LTCFs in these provinces with repeat assessments every 90 days. Therefore, these can be assumed to be population-level data for the participating provinces.

Table 1 provides a summary of the baseline characteristics by sleep disturbance status. Overall, two-thirds of the participants were female, over half were aged 85 years or older, and 61% had a dementia diagnosis. Baseline characteristics significantly differed between the two groups for all variables except for polypharmacy, chronic obstructive pulmonary disease (COPD), stroke and antipsychotics discontinuation. In general, individuals with sleep disturbance at baseline had higher prevalence rates or more severe symptoms for most conditions. For example, the prevalence of depression was 26.2% among individuals with sleep disturbance, compared to 22.3% in those without (*P* < 0.0001). Similarly, the prevalence of moderate to severe fatigue was 17.2% in those with sleep disturbance, compared to 13.8% in those without (*P* < 0.0001) (Table 1).

**Table 1:** Sample characteristics and distribution of sleep disturbance at baseline (T1)

Variable, %	Total sample (*N* = 21,394)	Sleep disturbance	
		No (*n* = 16,749)	Yes (*n* = 4645)
Gender			
Male	35.2	34.5	37.8
Female	64.8	65.5	62.2
Age			
65-74	14.4	14.3	15.0
75-84	31.8	31.3	33.5
85 +	53.8	54.5	51.5
Length of stay			
Less than 1 year	26.0	25.5	28.1
One year and more	74.0	74.5	71.9
Vision impairment			
None-minimal	85.7	86.4	83.5
Moderate-severe	14.3	13.6	16.5
Hearing Impairment			
None-minimal	81.5	82.2	78.7
Moderate-severe	18.5	17.8	21.3
Fatigue			
None-minimal	85.5	86.2	82.8
Moderate-severe	14.6	13.8	17.2
Cognitive Performance Scale (CPS)			
No impairment (0–1)	26.4	27.9	21.1
Mild-moderate impairment (2–3)	49.4	48.8	51.4
Severe impairment (4 +)	24.3	23.3	27.6
Activities of Daily Living Hierarchy Scale (ADL-H)			
No impairment (0–1)	19.7	20.3	17.5
Mild-moderate impairment (2–3)	43.8	43.0	46.9
Severe impairment (4 +)	36.5	36.8	35.7
Revised Index of Social Engagement (RISE)			
Low (0–2)	27.3	26.8	28.9
Moderate (3–4)	27.6	27.2	29.0
High (5–6)	45.2	46.0	42.1
Bladder incontinence	67.4	66.8	69.9
Bowel incontinence	51.0	50.3	53.4
Pain	11.4	10.1	16.1
Day time sleeping	32.7	32.1	35.1
Dementia	60.8	59.2	66.4
Parkinson's disease	5.7	5.4	6.9
Depression	23.1	22.3	26.2
Chronic obstructive pulmonary disease (COPD)	14.5	14.3	15.1*
Stroke	19.1	19.3	18.4*
Coronary Heart Disease (CHD)	19.5	18.4	23.6
Congestive Heart Failure (CHF)	14.3	14.1	15.3
Polypharmacy (9 or more medications)	40.0	39.7	41.1*
Antipsychotics (continued usage)	20.4	19.4	23.9
Antipsychotics (new usage)	4.2	3.9	5.3
Antipsychotics (discontinued)	2.6	2.5	2.9*
Hypnotics (continued usage)	13.3	12.0	18.2
Hypnotics (new usage)	5.8	5.4	7.2
Hypnotics (discontinued)	4.3	3.8	6.1

*P > 0.05

### The prevalence of sleep disturbance

**Fig. 1:**
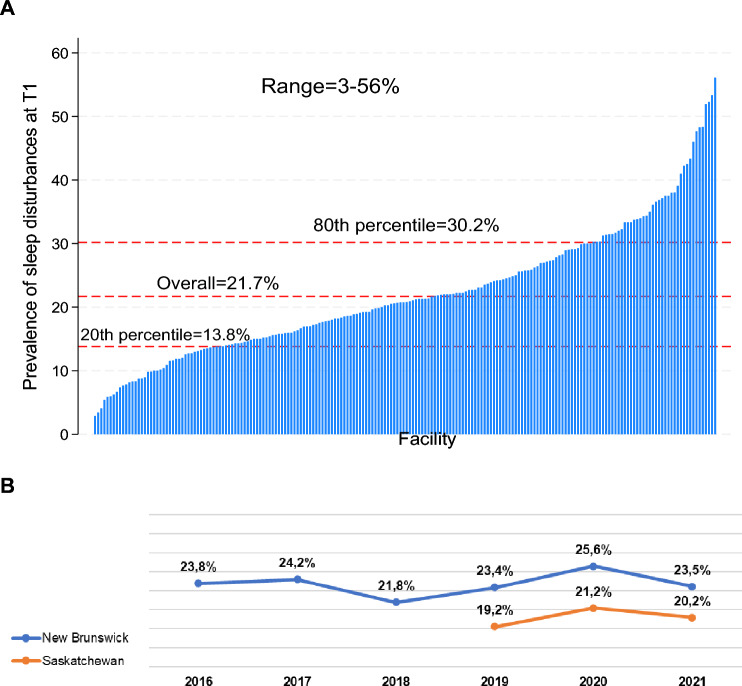
The prevalence of sleep disturbance across two Canadian provinces. **A** Bar graph of the prevalence of sleep disturbance across facilities in ascending order of prevalence. **B** Trend over time (2016–2021)

The overall prevalence of sleep disturbance at T1 was 21.7% (*n* = 4645). However, the prevalence ranged from 3 to 56% across facilities, with the 20th percentile at 13.8% and the 80th percentile at 30.2% (Fig. 1A). The prevalence of sleep disturbance remained consistent over the years in both provinces, although it was slightly elevated in 2020 during the COVID-19 pandemic (Fig. 1B).

Among the 16,749 residents without sleep disturbance at T1, 10.0% (*n* = 1675) experienced a worsening, developing sleep disturbance by T2. Conversely, among the 4,645 residents reporting sleep disturbance at T1, 51.9% (*n* = 2411) resolved, no longer experiencing sleep disturbance by T2.

### Predictors of new sleep disturbance

Table 2 presents the results of a multivariate GEE model identifying predictors of new sleep disturbance (model 1, *n* = 16,749). Nine variables were significantly associated with new sleep disturbance: male gender, a length of stay under one year, presence of pain, moderate cognitive impairment, daytime sleeping, COPD, coronary heart disease (CHD), antipsychotic medication use (continued, newly initiated, or recently discontinued), and sedative-hypnotic medication use (continued, newly initiated, or recently discontinued). For example, the adjusted odds of experiencing a new sleep disturbance CHD were 20% greater among residents with CHD compared to those without CHD (OR 1.20, 95% CI 1.06–1.36, *P* = 0.005). Similarly, the adjusted odds of experiencing new sleep disturbance among residents who initiated antipsychotic medications since the last assessment were 74% greater compared to those not using antipsychotics (OR 1.74, 95% CI 1.41–2.14, *P* < 0.0001).

**Table 2:** Predictors of new (model 1) and resolved (model 2) sleep disturbance

	New sleep disturbance (Model 1)				Resolved sleep disturbance (Model 2)			
	OR	95% CI		*P*	OR	95% CI		*P*
		Lower	Upper			Lower	Upper	
Female vs Male	0.90	0.82	0.99	0.023	1.08	0.96	1.22	0.181
Age (ref = 65–74)								
75–84	1.03	0.88	1.19	0.729	1.03	0.86	1.24	0.741
85+	1.04	0.89	1.20	0.643	1.00	0.83	1.19	0.976
CPS (Ref = no impairment)								
Mild-moderate impairment (2–3)	1.14	1.00	1.14	0.049	1.08	0.91	1.28	0.362
Severe impairment (4 +)	1.07	0.89	1.30	0.470	1.04	0.83	1.31	0.717
Pain (Ref = not present)	1.42	1.24	1.61	< 0.0001	1.18	0.99	1.41	0.069
Daytime sleeping (Ref = not present)	1.11	0.99	1.24	0.071	0.86	0.76	0.96	0.018
COPD (Ref = not diagnosed)	1.27	1.12	1.43	< 0.0.0001	0.87	0.75	1.02	0.084
Stroke (Ref = not diagnosed)	0.91	0.80	1.03	0.122	1.19	1.04	1.37	0.012
CHD (Ref = not diagnosed)	1.20	1.06	1.36	0.005	0.92	0.80	1.07	0.287
Polypharmacy (Ref = no)	0.92	0.83	1.03	0.165	1.17	1.02	1.35	0.025
Antipsychotics (Ref = Not used)								
Continued since the last assessment	1.21	1.06	1.37	0.004	1.02	0.88	1.19	0.789
Recently initiated	1.74	1.41	2.14	< .0001	1.02	0.79	1.32	0.899
Recently discontinued	1.44	1.05	1.98	0.022	0.99	0.69	1.42	0.973
Sedative-hypnotics (Ref = Not used)								
Continued since the last assessment	1.43	1.22	1.67	< .0001	0.71	0.60	0.85	0.000
Recently initiated	2.06	1.67	2.55	< .0001	0.72	0.58	0.89	0.003
Recently discontinued	1.61	1.27	2.03	< .0001	1.25	0.88	1.60	0.070
Length of stay (Ref = over 1 year)								
Less than 1 year	1.21	1.08	1.35	0.001	1.30	1.12	1.52	0.001

Table displays age, sex, and variables that demonstrated a significant association with the outcome measure in at least one of the models. While all variables listed in Table 1 were included in the multivariate analysis, only those with significant associations, along with age and sex, are reported in Table for clarity and ease of interpretation.*CPS* Cognitive Performance Scale, *COPD* Chronic Obstructive Pulmonary Disease, *CHD* Coronary Heart Disease.

### Predictors of resolved sleep disturbance

In model 2 (*n* = 4645), five factors were significantly associated with resolved sleep disturbance in the multivariate model: a length of stay under one year, daytime sleeping, stroke, polypharmacy and sedative-hypnotic medication use (continued or newly initiated). For example, the OR for daytime sleepers compared to non-daytime sleepers in the risk of resolved sleep disturbance was 0.86 (95% CI 0.76–0.96, *P* = 0.018), indicating that daytime sleepers had 14% lower odds of experiencing resolved sleep disturbance compared to those who did not sleep during the day. Similarly, new sedative-hypnotic medication users had 28% lower odds of having a resolved sleep disturbance compared to non-users (OR 0.72, 95% CI 0.58–0.89, *P* = 0.003). Interestingly, polypharmacy was positively associated with resolved sleep, with residents on polypharmacy having 17% greater odds of experiencing resolved sleep disturbance compared to those not on polypharmacy (OR 1.17, 95% CI 1.02–1.35, *P* = 0.025) (Table 2).

### Variance in the prevalence of sleep disturbance across facilities

**Fig. 2:**
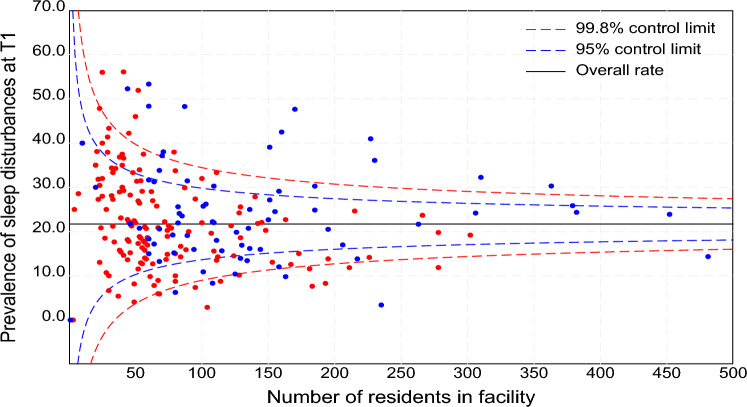
Funnel plots of the prevalence of sleep disturbance at T1. The circles represent individual facilities, and the solid line indicates the overall prevalence (the benchmark). The colour of the circles corresponds to provinces, with red representing facilities from Saskatchewan and blue from New Brunswick

Figure 2 presents the funnel plots illustrating significant variations in the prevalence of sleep disturbance across facilities. Of the 228 facilities, 8.3% (19 facilities) had prevalence rates above the upper 99.8% control limit, indicating an ‘alarm’ for high outlying rates. In contrast, 7.0% (16 facilities) had prevalence rates below the lower 99.8% control limit, suggesting significantly lower rates of sleep disturbance.

## Discussion

### Statement of principal findings

This study underscores the high prevalence of sleep disturbance in LTCFs, with one in five residents affected at baseline. Over half of those with sleep disturbance at baseline showed improvement over time, while 10% of those without sleep disturbance at baseline experienced new sleep disturbance. Prevalence rates varied widely across facilities, ranging from 3 to 56%, with 8% of facilities exhibiting unusually high rates. This unwarranted variation points to opportunities for targeted interventions to address these disparities. Importantly, the study identified several modifiable predictors of sleep disturbance, providing actionable insights for intervention strategies.

### Interpretation and comparison with existing literature

The prevalence of sleep disturbance in this study was 21.7%, falling within the broad range reported in previous studies conducted in LTCFs. Prior research has reported prevalence rates between 5 and 86% [4, 13, 14]. A systematic review and meta-analysis by Webster et al. examined the prevalence of sleep disturbance in LTCF residents with dementia across three scenarios: clinically relevant sleep disturbance measured using validated questionnaires (prevalence range 5–53%, pooled rate 20%), any sleep disturbance assessed via validated questionnaires (prevalence range 13–86%, pooled rate 38%), and sleep disturbance measured with actigraphy (prevalence range 32–84%, pooled rate 70%) [4]. In the German multicentre study, the overall prevalence of sleep disturbance (measured using the Sleep Disorder Inventory [15]) was 23%, with rates ranging from 0 to 85% across facilities [13]. These findings highlight the variability in reported prevalence rates due to differences in the methods used to assess sleep disturbance and the characteristics of the studied populations (e.g. age, comorbidities, and cognitive status). This discrepancy underscores the need for standardised measurement techniques and consistent definitions in future research to facilitate accurate comparisons across studies. Given the widespread implementation of interRAI assessments in multiple countries [16, 17], future cross-national studies utilising the interRAI data could facilitate rate comparisons and ensure the consistent application of risk adjustment methodologies, ultimately enhancing the validity and comparability of findings. For example, these standardised data were recently used in a 4-country study of influenza vaccination rates in LTCFs [18].

The prevalence of sleep disturbance remained consistently high over the years in both provinces, with a slight elevation observed in 2020 during the early phase of the COVID-19 pandemic. This pattern suggests that limited action has been taken to effectively address the issue. The modest increase during the early phase of the pandemic is particularly notable given the substantial disruptions to daily life and healthcare systems at that time. This could be attributed to pandemic-related factors such as increased anxiety, isolation due to visitor restrictions, and changes in daily routines [19]. These findings align with existing literature documenting the widespread mental health impacts of COVID-19 on older adults, particularly those in institutional care [20]. However, the lack of a marked increase suggests that care homes may have effectively mitigated some pandemic-related stressors or that other underlying factors in this population play a more dominant role in sleep disturbance. Future longitudinal studies could explore these nuances further.

Our findings revealed significant variation in the prevalence of sleep disturbance across facilities, with 8% exhibiting outlying rates indicative of unwarranted variation. However, it is important to emphasise that identifying outlying rates does not inherently indicate inappropriate care practices. Factors such as differences in resident case-mix, service models, facility characteristics, staffing levels, and access to specialist services likely contribute to the observed disparities. As highlighted by Karnon et al. [21] and DaSilva and Gray [22], identifying these variations can guide priority areas for further investigation and targeted interventions. These findings highlight the importance of conducting tailored, facility-specific reviews to uncover and address modifiable institutional and environmental factors contributing to these disparities. Insights from such reviews could inform the development of targeted, evidence-based interventions. Non-pharmacological strategies, such as cognitive-behavioural therapy for insomnia, daytime light therapy, and complementary practices like acupressure, have demonstrated efficacy in improving sleep outcomes [23, 24] and could be implemented to address these issues effectively.

Our study identified several modifiable risk factors for sleep disturbance, including pain, new admissions, daytime sleeping, and medications, alongside non-modifiable risk factors such as gender and specific health conditions. Pain, in particular, is a well-documented disruptor of sleep, and inadequate pain management can lead to insomnia and related issues [25]. Therefore, effective pain management is a critical component in reducing sleep disturbance. We note that the interRAI LTCF assessment measures pain frequency and severity in the context of current medication use, including analgesics.

In addition to pain, other modifiable and non-modifiable predictors warrant further discussion. For instance, older people with coronary heart disease (CHD) have been shown to have a higher likelihood of developing sleep disturbances [26]. This may reflect the bidirectional relationship between cardiovascular health and sleep quality, where poor cardiac function can lead to nocturnal symptoms such as dyspnoea, which disrupts sleep [27]. Similarly, cognitive impairment was associated with an increased risk of new sleep problems—likely due to disrupted circadian rhythms and behavioural symptoms such as nighttime wandering—while sleep disturbances may, in turn, exacerbate cognitive decline, indicating a bidirectional relationship [28, 29].

Daytime sleeping was associated with both the onset and persistence of sleep disturbance. This finding highlights the potential role of poor sleep hygiene and irregular sleep–wake patterns in LTCFs. Excessive daytime napping may result in decreased sleep drive at night, reinforcing a maladaptive sleep cycle. Strategies to promote structured daily routines and daytime engagement may help mitigate this issue. Furthermore, recent admission (length of stay < 1 year) was consistently associated with both the emergence and resolution of sleep problems. This dual association may reflect the transitional nature of early admission, where some residents experience adjustment-related sleep difficulties, while others benefit from stabilisation of routines and care in the facility [30]. Interventions aimed at supporting sleep during the admission period could be particularly impactful. Finally, stroke was positively associated with the resolution of sleep disturbance. Although this appears counterintuitive, one hypothesis is that residents post-stroke may receive more clinical attention or rehabilitation support, which indirectly improves sleep through better symptom control and structured routines. However, this warrants further investigation.

The association between medication use and sleep disturbance revealed complex patterns. Antipsychotic use—whether continued, newly initiated, or recently discontinued—was significantly associated with new sleep disturbance, which is consistent with some previous studies [31, 32]. This finding may reflect the sedative but paradoxically disruptive effects of antipsychotics on sleep architecture, particularly in older adults. It may also reflect attributes of residents not fully controlled in these analyses that lead to both antipsychotic use and sleep disturbance.

Sedative-hypnotic medications, a class of drugs commonly prescribed for anxiety and sleep-related disorders, were negatively associated with sleep disturbance in both models. Their use was not linked to the resolution of sleep disturbance nor the prevention of new sleep disturbance. As with antipsychotic use noted above, this pattern may reflect *confounding by indication*, as individuals prescribed these medications were more likely to have had severe sleep disturbance that did not fully respond to treatment. The observed lack of benefit could have been due to the underlying severity of sleep issues or the limited effectiveness of the medication in resolving them. Additionally, the influence of sedative-hypnotics on sleep health may be further shaped by underlying medical conditions that impact sleep quality. While sedative-hypnotics may provide short-term relief, evidence suggests they pose significant long-term risks, including dependency, rebound insomnia, and other adverse health outcomes [33, 34]. The short-term benefits of sedative-hypnotic medications must be carefully weighed against their potential long-term harms, considering residents’ overall health and quality of life. It is also important to recognise that most sedative-hypnotics are classified as *potentially inappropriate medications* for older adults under the 2023 updated Beers Criteria, a guideline supported by high-quality evidence [35]. Consequently, their use for managing sleep disturbance in LTCFs is generally discouraged [36]. Clinicians should approach these medications on a case-by-case basis, ensuring that their use aligns with individualised care goals and prioritises non-pharmacological interventions whenever feasible. In future research, it would be helpful to employ treatment effects analyses to more fully control for the problem of confounding by indication as has been done with interRAI data on antipsychotic use and behaviour disturbance [37].

The association between polypharmacy and resolved sleep disturbance presents an intriguing finding. While polypharmacy is generally linked to adverse outcomes [38], including increased risk of drug interactions and side effects, the observed association with sleep disturbance resolution may reflect oversedation or CNS depression resulting from the use of multiple medications. Alternatively, residents receiving multiple medications benefited from more comprehensive care plans, including targeted interventions for sleep disturbance.However, this finding should not be interpreted as an endorsement of excessive medication use. The risks associated with polypharmacy, especially in frail older patients, such as cognitive impairment, falls, and reduced quality of life, underscore the critical need for careful medication management. This counterintuitive finding warrants further investigation to determine whether polypharmacy is associated with increased daytime sleeping as well as changes in nighttime sleep patterns.

### Implications for practice and policy

The observed associations between medication use and sleep disturbance emphasise the critical role of optimising pharmacological management in LTCFs. Efforts to reduce inappropriate antipsychotic use and carefully monitor sedative-hypnotic medications should be integrated into routine care practices [39]. Policymakers should consider implementing guidelines that promote deprescribing when clinically appropriate [40], alongside strategies to support non-pharmacological interventions.

These results also point to opportunities to enhance the use of interRAI systems for improving quality of care. For example, although interRAI’s mental health assessments include care planning protocols for sleep disturbance an equivalent is not yet available for the LTCF [41]. The present results suggest that triggering rates for the sleep items are consistent with those reported in the literature and the LTCF contains a number of risk factors that should be considered in the development of a person-centred care plan. In addition, given the facility-level variations in sleep disturbance and the availability of appropriate covariates for risk adjustment, consideration should be given to the development of a risk-adjusted quality indicator for sleep that can be used for internal quality improvement and for public reporting, as has been done with other interRAI indicators [42, 43].

### Strengths and limitations of the study

The strengths of the study include its large representative sample size, a longitudinal design and the inclusion of data from multiple facilities across two provinces. The use of a standardised data collection tool ensures consistency, accuracy, and efficiency, enhancing comparability and reducing bias. However, some limitations warrant consideration. The observational nature of the study precludes causal inference, and residual confounding by unmeasured factors cannot be ruled out. Data on individual-level factors, such as the use of non-pharmacological interventions for managing sleep disturbance, were limited, potentially influencing our findings.

Our dataset lacked facility-level data, such as staffing ratios and environmental factors (e.g. noise, lighting, room arrangements), which may affect sleep quality and contribute to inter-facility variability. While facility size could be estimated, our analysis focused on person-level risk factors, and these unmeasured factors may have contributed to residual confounding.

We used a single-item measure of sleep disturbance from the interRAI LTCF, which has not been validated against gold-standard measures such as polysomnography or the Pittsburgh Sleep Quality Index [44]. However, obtaining criterion validity against gold-standard measures such as polysomnography is neither practical nor ethical in nursing home populations, given the invasiveness and burden for frail residents. Nonetheless, the interRAI sleep item has demonstrated several forms of validity. Face and content validity were established through extensive international consultation with experts and frontline clinicians, supported by literature reviews informing item design. Convergent validity is supported by multiple studies showing consistent associations between sleep disturbance and related outcomes, including distressed mood, across over 500,000 individuals in diverse care settings [45], links with fatigue and depression in older adults [46], and prediction of caregiver distress in a large New Zealand cohort [47]. While predictive validity has yet to be fully explored, particularly for outcomes such as daytime drowsiness or fatigue, these findings provide strong support for the construct validity of the sleep item in long-term care contexts.

For persons who are unable to respond there is potential for misclassification due to reliance on staff assessments alone. We did not present risk-adjusted rates in our funnel plots showing variations in the prevalence of sleep disturbance across facilities. As a result, the observed variability may be partly explained by differences in resident characteristics and underlying health conditions, which were not adjusted for in our analysis. Finally, our medication data were limited to class-level indicators (antipsychotics, antidepressants, anxiolytics, and sedative-hypnotics), as detailed drug-level information, including timing and dosage, was unavailable, which may have introduced residual confounding.

## Conclusion

This study highlights the high prevalence of sleep disturbance in LTCFs and significant variations across facilities, with potential modifiable risk factors offering opportunities for improvement. Medication-related factors, particularly the association of antipsychotics and sedative-hypnotic medications use with sleep disturbance, underscore the need for careful prescribing practices. Future research should explore the complex interplay of institutional, environmental, and individual factors influencing sleep disturbance, to develop tailored interventions that enhance the quality of care in LTCFs.

## Supplementary Information

Below is the link to the electronic supplementary material.

Supplementary file1 (DOCX 50 kb)

## Data Availability

The data that support the findings of this study are available on request from the corresponding author. The data are not publicly available due to privacy or ethical restrictions.

## Code availability

The code that supports the findings of this study is available upon reasonable request from the corresponding author.
