# Supplementary figures and images for: Predictors of change in sleep disturbance in Canadian long-term care facilities: a longitudinal analysis based on interRAI assessments
Source: Eur Geriatr Med. 2025 Sep 10;16(6):2281–91. doi: 10.1007/s41999-025-01302-z (PMC12743656; doi:10.1007/s41999-025-01302-z)

# Supplementary File


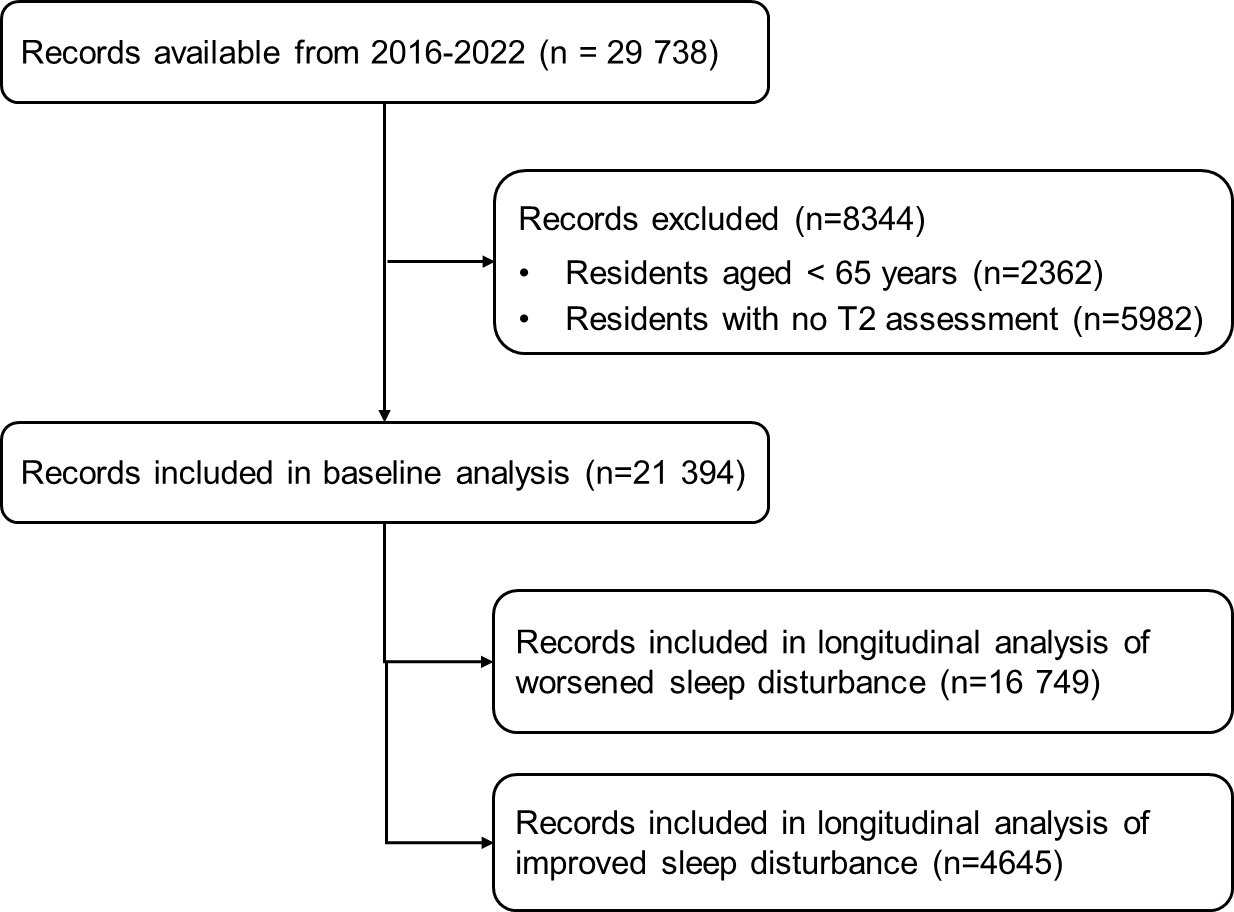


Figure 1: Participants selection criteria.

Supplement: Supplementary file 1 — Supplementary file1 (DOCX 50 kb) [file 41999_2025_1302_MOESM1_ESM.docx]
